# New Aspects of Mitochondrial Uncoupling Proteins (UCPs) and Their Roles in Tumorigenesis

**DOI:** 10.3390/ijms12085285

**Published:** 2011-08-17

**Authors:** Delira Robbins, Yunfeng Zhao

**Affiliations:** Department of Pharmacology, Toxicology & Neuroscience, LSU Health Sciences Center 1501 Kings Highway, Shreveport, LA 71130, USA; E-Mail: drobbi@lsuhsc.edu

**Keywords:** mitochondrial uncoupling, UCP2, cancer, UCP2 regulation

## Abstract

Uncoupling proteins (UCPs) belong to a family of mitochondrial carrier proteins that are present in the mitochondrial inner membrane. UCP1 was first identified followed by its two homologs, UCP2 and UCP3. The physiological functions of UCP include lowering mitochondrial membrane potential and dissipating metabolic energy as heat. However, UCP can be dysregulated and may contribute to the pathogenesis of metabolic disorders and obesity. Recent studies suggest that UCP also plays a role in neurodegenerative diseases and atherosclerosis. In addition, the widely expressed UCP, UCP2, has been shown to be upregulated in a number of aggressive human cancers. One mechanism of UCP2 upregulation in these cancers is due to oxidative stress, and elevated UCP2 in turn reduces oxidative stress, which provides a growth advantage for these cancers. Nevertheless, new studies suggest UCP2 may interact with oncogenes and tumor suppressor genes, providing a potential new mechanism of how UCP2 contributes to cancer development. In this review, the evidence supporting the role of UCPs in diseases other than diabetes and obesity, the reports on how UCP is regulated in cancer cells, and how UCP may regulate p53 will be discussed.

## 1. Introduction

In vertebrates, energy is mainly produced in the mitochondria via the Citric Acid Cycle coupled with oxidative phosphorylation. The mitochondrial respiration process generates a proton gradient across the mitochondrial inner membrane that establishes the electrochemical potential (Δ*ψ*_m_), which is mainly used for ATP synthesis. However, not all of the energy available in the electrochemical gradient is coupled to ATP synthesis. Some of the energy is consumed by “proton leak” reactions, by which protons pumped into the inner membrane space flow back into the matrix through proton conductance pathways in the inner membrane that bypass the ATP synthase. As a result, energy derived from the metabolic oxidation reaction is dissipated as heat [1–4]. This nonproductive proton leak, termed mitochondrial uncoupling, is physiologically important and accounts for 20–25% of the basal metabolic rate [5,6].

Mitochondrial uncoupling is mediated mainly by uncoupling proteins (UCPs), among those, UCP1 is the first to be identified in brown adipose tissue [7], followed by its four homologs: UCP2 [8] is ubiquitously expressed, UCP3 [9] exists solely in skeletal muscle and the heart, UCP4 [10], and BMCP1 (brain mitochondrial carrier protein-1, [11]) or UCP5 are predominantly expressed in the central nervous system. UCPs are anion carriers across the mitochondrial inner membrane, which bring protons back into the mitochondrial matrix. In addition, UCP1 dissipates redox energy and thereby provides heat to the animal. UCP2 decreases the production of reactive oxygen species [12]. Furthermore, it has been suggested that brain-specific UCP4 and UCP5 play a role in apoptosis in the brain [13,14].

## 2. UCPs and Non-Cancer Diseases

Mitochondria-generated ATP provides the major fuel for eukaryotic cells. As byproducts, free radicals can be generated within mitochondria during energy production, which could harm cells if not rapidly removed. Mild mitochondrial uncoupling can reduce the generation of free radicals and therefore, protect cells. Mitochondrial uncoupling has been amplified or reduced during the pathogenesis of a number of human diseases due to up- or down-regulation of UCP. The two most studied UCP-associated diseases are diabetes and obesity, and studies of UCP in these two diseases have been thoroughly reviewed.

Non-alcoholic fatty liver disease is another form of metabolic disorder. Park *et al*. detected UCP2 in young Asian patients, and the results showed that the expression levels of UCP2 were correlated with general pathologic severity [15].

Non-alcoholic steatohepatitis (NASH) has been identified in patients with non-alcoholic fatty liver disease with evidence of inflammation in liver biopsies. The mechanistic progression of non-alcoholic fatty liver disease to non-alcoholic steatohepatitis is not clear; however the involvement of underlying mitochondrial dysfunction orchestrates the progression due to its role in fatty acid oxidation, ROS generation and ATP synthesis. NASH has been characterized by the accumulation of fatty acids in hepatocytes and oxidative stress [16], decreased ATP production [17], and the induction of proinflammatory cytokines [18]. Serviddio *et al*. reports that the upregulation of UCP2 protects the liver from excessive fat accumulation, but chronically depletes the liver of ATP, compromising the response of the liver to acute energy demands, which results in increased susceptibility to ischemia-reperfusion injury [19].

Clinical evidence suggests that UCPs may be associated with diseases distinct from metabolic diseases. For instance, higher expression levels of UCP2 may mediate follicle development in polycystic ovary syndrome [20]. Several well designed studies further suggest that UCP is involved in the pathogenesis of neurodegenerative disease, atherosclerosis, liver disease, *etc*.

Richard *et al*. reported the presence of UCP2 mRNA with varying intensities throughout the brain. Marked high intensities were found in the hypothalamus, the ventral septal region, the ventricular region and the cerebellum, suggesting a novel role of UCP in modulating neuroendocrine functions and autonomic responses of the brain [21]. In addition, UCP2-mediated mitochondrial proton leak has been positively correlated with increased oxygen consumption in brain tissues [22].This regulation of oxygen consumption, suggests that UCP2 may modulate ROS production and influence the process of neurodegeneration [21].

The mRNA expression of UCP2 in the brain suggested neuronal localization [21]. In an animal model of Parkinson’s disease (PD), in which dopamine neurons are depleted by 1-methyl-4-phenyl-1,2,5,6 tetrahydropyridine (MPTP), UCP2 knockout increased whereas UCP2 overexpression decreased MPTP-induced nigral dopamine cell loss. Coenzyme Q_10_ (CoQ_10_), a cofactor of mitochondrial metabolism and UCP2 activity [23], induced UCP2-mediated mitochondrial uncoupling in the substantia nigra [24]. When given orally, CoQ_10_ was shown to reduce dopamine cell loss in both mouse [25] and primate models of PD [24] and potentially slowed the progression of the disease in human patients [26]. As mentioned above, UCP2 protects against dopamine cell loss caused by the mitochondrial complex I toxin MPTP. Consistent with that, degeneration of substantia nigra compacta dopaminergic neurons in human PD is associated with mitochondrial complex I dysfunction and free radical toxicity [27]. These studies demonstrate the importance of UCP2 in normal nigral dopamine cell metabolism, and suggest that UCP2 is important in regulating cell survival and susceptibility to mitochondrial toxins; and may serve as a novel therapeutic target for the prevention and treatment of PD [28].

Not surprising, UCP also plays an important role in Alzheimer’s disease (AD). The expression levels of UCP2, 4, and 5 were significantly reduced in AD patients, which were accompanied by upregulation of nitric oxide synthases (NOS), and resulted in increased oxidative stress and impaired mitochondrial functions. Therefore, to increase the expression level of UCP may help in the treatment of AD [29].

The protective role of UCP2 against atherosclerosis was demonstrated using irradiated low-density lipoprotein receptor-deficient mice (*LDLR*-/-). These mice were transplanted with bone marrow from either UCP2 deficient mice or wild-type mice, and fed with an atherogenic diet. The results showed that the atherosclerotic lesion size was markedly increased in the mice receiving UCP2 deficient bone marrow, which was accompanied by enhanced oxidative stress [30].

## 3. UCP and Cancer

Compared with the extensive studies on UCP in diabetes and obesity, the role of UCP (mostly UCP2) in cancer was recently recognized and has attracted more attention. As a matter of fact, the impact of mitochondrial uncoupling on cellular physiology is not restricted to normal cells; it also plays an important role in the reprogramming of cancer cell metabolism [31].

Early evidence showing that UCP might be important for tumor growth arises from rats which received an inoculation of the Yoshida AH-130 ascites hepatoma cells [11]. The expression levels of both UCP2 and UCP3 mRNAs were increased, suggesting that energy expenditure is associated with tumor growth.

In human studies, most of the results point to UCP2 potentially being associated with the development of colon and breast cancer. Horimoto *et al*. reported that UCP2 overexpression in malignancy was limited to the colon [32], in which UCP2 expression in human colon tissue increased with the degree of neoplasia. However, their results also showed that high UCP2 levels in colon adenocarcinoma correlated with high oxidant levels, making it hard to explain whether it is a cause or effect relationship between uncoupling and oxidative stress.

Kuai *et al*. further demonstrated that the levels of both UCP2 mRNA and protein were higher in colon cancer tissue samples than in its adjacent tissue samples [33], and the authors suggested that increased UCP2 expression may be involved in colon cancer metastasis.

However, the first colon tumorigenesis study using UCP2 null mice showed surprising results [34]. The UCP2 null mice developed more colon tumors than the wild-type controls with increased oxidative stress and enhanced NF-κB activation when treated with azoxymethane (AOM), an experimental alkylating carcinogen [35], despite the fact that isolated mitochondria from UCP2-/- cells produced more superoxide/hydrogen peroxide. In our opinion, this study can be improved by checking whether UCP2 is up- or down-regulated during colon tumorigenesis in the wild-type mice. If it is upregulated, then UCP2 transgenic mice might be a better tool to study the role of UCP2 in colon tumorigenesis.

Later studies suggest that UCP2 is also involved in the pathogenesis of other cancers. Sayeed *et al*. reported a significant association of UCP2 with tumor grade in primary breast cancer [36]. The results are reinforced by the study from Won *et al*., which demonstrated that UCP2 expression was correlated significantly with histological grade and mitotic count in invasive ductal carcinoma of the breast analyzed from human tissue microarrays [37].

We used a tumor promotion model to study the role of mitochondrial uncoupling in tumor promotion [38]. Our study demonstrated that mitochondrial uncouplers inhibited cell death that arose during tumor promotion. When UCP2 was downregulated using a siRNA approach, cell transformation was suppressed, suggesting UCP2 may serve as a tumor promoter during early tumorigenesis.

Another important role of UCPs in cancer is their contributions to chemoresistance [39,32]. UCPs were upregulated in many chemoresistant cancer cell lines, which may provide a prosurvival advantage to tumor cells via attenuating ROS (reactive oxygen species) generation [40–42], and increasing drug concentrations to kill these cancer cells [43].

## 4. Regulation of UCP in Cancer Cells

UCP gene regulation is not well understood. UCP can be generally regulated by oxidative stress. When oxidative stress occurs, UCP2 and UCP3 uncoupling activities can be stimulated by superoxide anion, limiting ROS production by the mitochondrial respiratory chain. This in turn decreases superoxide levels and UCP uncoupling activity via a feedback loop [44]. Fatty acids also induced UCP2 and UCP3 in muscle [45] and fat [46], which was mediated by transcription factors of peroxisome proliferator-activated receptors (PPAR, [47]).

How oncogenes and tumor suppressor genes may regulate UCP should be of great interest to study UCP in cancer. However, such evidence is rare, which deserves more research efforts, since knowing the basic biology of UCP regulation in cancer cells is essential for potential UCP-targeted cancer therapy. One important study utilized well-to-moderately differentiated primary breast tumor cell lines showed that SMAD was recruited to the UCP2 promoter region via the transforming growth factor-beta (TGFβ)/SMAD-dependent pathway [36]. In contrast, in TGFβ-resistant high-grade tumors, potentiation of UCP2 repression fails to occur, contributing to impaired cell differentiation.

## 5. UCP2 and p53 Signaling

p53, as a pivotal tumor suppressor, can cause apoptotic cell death in response to cellular stress stimuli (e.g., drugs, irradiation, UV, hypoxia) and the expression of viral or cellular oncogenes [48]. Interestingly, a fraction of p53 can translocate to mitochondria under the above mentioned stress conditions [49]. Similarly to nuclear p53, mitochondrial p53 is also able to initiate apoptosis [50]. Since both p53 and UCP2 can regulate and respond to oxidative stress, it is possible that there is a potential interaction between these two proteins.

A few studies have suggested that this may indeed occur. Derdak *et al*. demonstrated that UCP2 inhibited apoptosis of colon cancer cells by suppressing the phosphorylation of p53 within the transactivating domain via suppressing ROS [43]. Won *et al*. had a different observation. They reported that high UCP2 expression in invasive breast ductal carcinoma was associated with high p53 nuclear expression [37]. However, the antibody against p53 used (DO-7) can detect both wild-type and mutant p53, and p53 is highly mutated in these tumors [51]. Therefore, it is not clear if the highly expressed p53 was wild-type or mutant or both.

Our studies using the skin carcinogenesis model revealed that during early tumor promotion, the tumor suppressor p53 translocated to mitochondria and physically interacted with a primary antioxidant defense enzyme, manganese superoxide dismutase (MnSOD), leading to suppression of its superoxide scavenging activity, as well as, increases in ROS levels [52]. Thus, in addition to the direct apoptotic activity of mitochondria p53, the ability to induce ROS accumulation might serve as a positive feed-back loop and play an essential role in the p53-mediated apoptosis pathway.

Given these facts, we further examined the role of UCP2 in tumor promoter-induced p53 mitochondrial translocation and cell transformation. Downregulation of UCP2 expression using a transfection with specific siRNA to UCP2 induced translocation of a small amount of p53 to mitochondria, and also enhanced tumor promoter-induced p53 mitochondrial translocation whereas cell transformation was reduced [38]. These data indicated that UCP2-mediated mild mitochondrial uncoupling may serve as a tumor promoting event.

The elevated levels of UCP2 in cancer cells may be a result of long-term selection during tumorigenesis, since any event that results in UCP2 upregulation could help cells escape from apoptosis mediated by the p53 signaling. Given the fact that mitochondrial uncoupling could cause dissipation of the mitochondrial potential, a decrease in mitochondrial ROS, and a reduction in p53’s response to oxidative stress, it is reasonable to propose that mitochondrial uncoupling may provide cancer cells with a prosurvival advantage via suppressing the p53 mediated apoptosis pathway.

## 6. Conclusions

It has been suggested that dysregulation of UCP is involved in the pathogenesis of not only diabetes and obesity, but also neurodegenerative disease, atherosclerosis, and cancer. Among these diseases, it is very likely that UCP can protect against neurodegeneration, but exacerbate the progression of the other diseases. In cancer, elevated UCP contributes not only to chemoresistance, but also to early transformation. Therefore, targeting UCP could serve as a promising approach for both cancer prevention and therapy.
